# Supplementary material for: Cryo-EM elucidates the uroplakin complex structure within liquid-crystalline lipids in the porcine urothelial membrane
Source: Commun Biol. 2023 Oct 7;6:1018. doi: 10.1038/s42003-023-05393-x (PMC10560298; doi:10.1038/s42003-023-05393-x)
Supplement: Supplementary file 3 — Description of Additional Supplementary Files [file 42003_2023_5393_MOESM3_ESM.pdf]

Description of Additional Supplementary Files

File name: Supplemental Data 1
Description: Maps and models used in this manuscript.

File name: Supplemental Data 2
Description: Numerical data used for generating boxplot in Figure 6.

File name: Supplemental Movie 1
Description: Movie representation of Figure 2A.
